# Cooperativity of membrane-protein and protein–protein interactions control membrane remodeling by epsin 1 and affects clathrin-mediated endocytosis

**DOI:** 10.1007/s00018-020-03647-z

**Published:** 2020-09-30

**Authors:** Benjamin Kroppen, Nelli Teske, King F. Yambire, Niels Denkert, Indrani Mukherjee, Daryna Tarasenko, Garima Jaipuria, Markus Zweckstetter, Ira Milosevic, Claudia Steinem, Michael Meinecke

**Affiliations:** 1 Department of Cellular Biochemistry, University Medical Center Göttingen, Humboldtallee 23, 37073 Göttingen, Germany; 2 Institute for Organic and Biomolecular Chemistry, University of Göttingen, Tammannstr. 2, 37077 Göttingen, Germany; 3 European Neuroscience Institute, Göttingen – A Joint Initiative of the University Medical Center Göttingen and the Max-Planck-Society, Grisebachstr. 5, 37077 Göttingen, Germany; 4 German Center for Neurodegenerative Diseases (DZNE), Von-Siebold-Str. 3a, 37075 Göttingen, Germany; 5 Max Planck Institute for Biophysical Chemistry, Am Fassberg 11, 37077 Göttingen, Germany; 6 Göttinger Zentrum für Molekulare Biowissenschaften – GZMB, 37077 Göttingen, Germany; 7 Max Planck Institute for Dynamics and Self-Organization, Am Faßberg 17, 37077 Göttingen, Germany; 8 Present Address: Wellcome Centre for Human Genetics, Nuffield Department of Medicine, NIHR Oxford Biomedical Research Centre, University of Oxford, Oxford, OX3 7BN UK

**Keywords:** Membrane curvature, Membrane dynamics, ENTH domain, Clathrin-mediated endocytosis, Reconstitution of membrane dynamics

## Abstract

Membrane remodeling is a critical process for many membrane trafficking events, including clathrin-mediated endocytosis. Several molecular mechanisms for protein-induced membrane curvature have been described in some detail. Contrary, the effect that the physico-chemical properties of the membrane have on these processes is far less well understood. Here, we show that the membrane binding and curvature-inducing ENTH domain of epsin1 is regulated by phosphatidylserine (PS). ENTH binds to membranes in a PI(4,5)P_2_-dependent manner but only induces curvature in the presence of PS. On PS-containing membranes, the ENTH domain forms rigid homo-oligomers and assembles into clusters. Membrane binding and membrane remodeling can be separated by structure-to-function mutants. Such oligomerization mutants bind to membranes but do not show membrane remodeling activity. In vivo, they are not able to rescue defects in epidermal growth factor receptor (EGFR) endocytosis in epsin knock-down cells. Together, these data show that the membrane lipid composition is important for the regulation of protein-dependent membrane deformation during clathrin-mediated endocytosis.

**Electronic supplementary material:**

The online version of this article (10.1007/s00018-020-03647-z) contains supplementary material, which is available to authorized users.

## Introduction

Clathrin-mediated endocytosis (CME) is a special form of vesicle budding from the plasma membrane that capitalizes on a formation of clathrin lattices around maturing buds. In this orchestrated and highly dynamic process some 50 proteins come together to internalize receptors, extracellular ligands or to recycle synaptic vesicles [1, 2]. Over the course of several seconds the plasma membrane undergoes heavy remodeling. A clathrin-coated pit is formed and eventually pinched of the plasma membrane to release a highly curved clathrin-coated vesicle (CCV).

Over the years a number of proteins with the ability to deform membranes were identified to be important in this process [3, 4]. One family of these proteins are epsins. Epsins are evolutionary conserved proteins, which in mammals consist of three genes, epsin 1–3 [5]. Epsin1 consists of a long, unfolded, C-terminal protein–protein interaction domain and an N-terminal membrane binding domain. With the N-terminal ENTH domain (epsin N-terminal homology), epsin1 binds to the cytoplasmic surface of the phosphatidylinositol-4,5-bisphosphate (PI(4,5)P_2_)-containing plasma membrane. Epsin was shown to be involved in CME, especially in the uptake of epidermal growth factor (EGF) [6]. It has also been implicated in CME of other cargos like transferrin, but here its role is less well understood [6, 7]. Upon PI(4,5)P_2_ binding a formerly unstructured region at the very N-terminus folds into an amphipathic helix, called helix 0 [8, 9]. This amphipathic helix inserts into the membrane, which probably induces membrane curvature, which is believed to be important to drive clathrin-coated pit formation [10]. Despite the necessity of PI(4,5)P_2_ to bind the ENTH domain, almost nothing is known about the involvement of the membrane lipid composition in protein-dependent membrane deformation.

In this study we have identified the inner leaflet plasma membrane lipid phosphatidylserine (PS) to be crucial for the membrane curvature-inducing activity of the ENTH domain. Presence of this lipid triggers homo-oligomerization of the epsin protein, and through structure–function analysis we show that this assembly is important for membrane deformation.

## Methods

### Protein biochemistry

#### Protein expression and purification

The ENTH domain and its mutants were recombinantly expressed in *E. coli* BL21(DE3) cells (Stratagene, California, USA). Therefore, the cells were transformed by heat shock with pGEX-6P-2 vectors (including genes for Rat Epsin1 WT ENTH 1–164 and generated mutants). The recombinant expression was performed by isopropyl-β-D-thiogalactopyranoside (IPTG) induction. Transformed, selected, single colonies of *E. coli* BL21(DE3) were transferred into LB medium (10 g/l NaCl, 5 g/l yeast extract, 10 g/l tryptone, 0.1 g/l ampicillin) and cultivated for about 2.5 h at 37 °C and 140 rpm agitation until reaching an optical density (OD_600_) of 0.6–0.8. The expression culture was supplemented with 1 mM IPTG and incubated for 3 h at 30 °C and 140 rpm agitation. The expression culture was finally centrifuged for 10 min at 4 °C and 5316×*g* (Thermo Scientific H-12000 BioProcessing Rotor). The cell pellet was resuspended in 40 ml HEPES buffer per 2 l culture on ice. The cell suspension was subsequently centrifuged for 30 min at 4 °C and 3250×*g* (Eppendorf Swing-bucket rotor A-4-62). The supernatant was discarded. To obtain single-band pure ENTH domain a three-step chromatographic purification strategy was performed. In the first step the ENTH domain was isolated from the soluble cell lysate as GST fusion protein using GSTrapFF™ 5 ml columns together with the ÄKTAPrime Plus system (GE Healthcare Life Science, Chalfont St Giles, UK). The cell lysate was applied onto the column (0.4 ml/min, 8 °C) and non-binding fractions were separated by washing with 25 ml GST binding buffer (5 ml/min, 8 °C). The target protein was eluted with GST elution buffer (1 ml/min, 8 °C). The GST-tag was proteolytically cleaved from the ENTH domain using PreScission Protease with a molar ratio of 1:100 (C_fusion protein_: C_PreScission Protease_). The digest was performed for 16 h at 8 °C with gentle agitation.

To separate the ENTH domain from the cleaved GST-tag an anion exchange chromatography was performed using HiTrap™ Q HP, 5 ml (GE Healthcare Life Science, Chalfont St Giles, UK). The protein mixture was loaded onto the column using anion exchange low salt buffer (50 mM NaCl, 50 mM HEPES/NaOH, pH 8.0) at a flow-rate of 1 ml/min at 8 °C. The free ENTH domain eluted during the washing step with the same buffer (3 ml/min, 8 °C) while GST remained bound to the column until using anion exchange high salt buffer (1 M NaCl, 50 mM HEPES/NaOH, pH 8.0). A size-exclusion chromatography column HiLoad™ 16/600 Superdex™ 75 pg (GE Healthcare Life Science, Chalfont St Giles, UK) was used in the final step to ensure the purity of the ENTH domain. The ENTH domain was eluted with HEPES buffer (or PBS buffer if it was labeled afterwards) at 1 ml/min and 8 °C. The proteins were directly used afterwards or stored at -80 °C.

#### Visualization of LUV deformation by electron microscopy

Large unilamellar vesicles were prepared as described before [11, 12]. Briefly, L-α-phosphatidylcholin (PC), L-α-phosphatidylethanolamine (PE), L-α-phosphatidylserine (PS) and L-α-Phosphatidylinositol-4,5-bisphosphate (PIP_2_) were obtained from Avanti Polar Lipids (Alabaster, AL).

To observe the protein-induced deformation of LUVs (as described before [13, 14]), liposomes (0.2 mg/ml) were incubated with ENTH domain or it’s mutants (15 µM) at 30 °C for 3 h. The samples were subsequently diluted to 0.2 mg/ml of liposomes with HEPES buffer (200 mM NaCl, 10 mM HEPES/NaOH, pH 7.4) and 5 µl of the suspension was then transferred onto a formvar carbon coated copper grid (Agar Scientific Ltd., Essex, UK) and incubated for 1 min at room temperature. The suspension was removed and the grid was set onto a droplet (50 µl) of 3% uranyl acetate for negative staining.

Electron microscopic visualization was performed with a JEOL JEM 1011 transmission electron microscope (JEOL Ltd., Akishima, Japan) and a Gatan Orius 1000 CCS detector (Gatan Inc., Pleasanton, USA).

#### Binding and membrane deformation dynamics on GUVs

GUVs were prepared as previously described with 0.5% of the fluorescently labeled lipid Atto647N PtdEnt (ATTO-TEC GmbH, Siegen, Germany). 50 µl of the GUV suspension was carefully transferred into the microscopy chamber Nunc® Lab-Tek® II chambered coverglass (Thermo Fisher Scientific Inc., Waltham, USA), that was coated with lipid-free bovine serum albumin (BSA, Sigma-Aldrich Crop., St. Louis, USA) and filled with 250 µl of GUV buffer (192 mM NaCl, 10 mM HEPES/NaOH, pH 7.4).

The fluorescence spinning disc confocal microscopy was performed at 25 °C with a Nikon Eclipse Ti microscope (Nikon Instruments K.K., Tokyo, Japan), a PerkinElmer UltraVIEW VoX system (Perkin Elmer, Waltham, USA) with a spinning disk confocal scan head CSU-X1 (Yokogawa Denki K. K., Tokyo, Japan) and an Electron Multiplying CCD Camera C9100 (Hamamatsu Photonics K.K., Hamamatsu, Japan). The data was acquired with the software Volocity® 6.3 (PerkinElmer Corp., Waltham, USA). Proteins that were labeled with Atto488 maleimide (ATTO-TEC GmbH, Siegen, Germany) were added to the GUV suspension in 10 µM steps. The fluorophores were excited at the optimal wavelength and detected at the optimal emission wavelength according to the manufacturer (ATTO-TEC GmbH, Siegen, Germany). All fluorophores were excited at low laser powers (5–8%) and at fast exposure times (50–120 ms). Images were taken with a speed of 30 frames per minute for 30 min.

#### Secondary structure analysis by CD spectroscopy

Circular dichroism (CD) spectroscopy was used to compare the folding of the wild-type ENTH domain and the ENTH domain mutant R114A using a Chirascan CD Spectrometer (Applied Photophysics Ltd., London, UK). Proteins were transferred into a 10 mM sodium potassium phosphate buffer pH 7.4 with a concentration of 0.1 mg/ml. The measurements were carried out at 25 °C between 180 and 260 nm with 1 nm step sizes and an accumulation time of 5 s. The mean residue weight ellipticity was calculated according to Kelly S. M. et al. ^188^ with the following formula:

$$\left[ \Theta \right]_{{{\text{MRW}}}} \, = \,(\Theta \cdot {\text{MRW}}) \, / \, ({1}0 \cdot c \cdot d).$$

The molar ellipticity *Θ* and the mean residue weight (MRW) are dependent on the optical path length *d* and the protein concentration *c*.

#### Co-sedimentation assay (Spin assay) 

The co-sedimentation assay (spin assay) was used for two purposes:

1. To detect protein binding to LUVs by co-sedimentation.
2. To detect if small vesicles were generated due to membrane deformation. The sedimentation of spherical particles in aqueous solution is described by Stokes’s law.

The sedimentation behavior of a liposome is more dependent on the size than on the density and it is possible to separate SUVs from LUVs by centrifugation.

LUVs were prepared as described before, diluted to 2 mg/ml and mixed with 20 µM purified proteins. After incubating the sample for 3 h at 30 °C, 150 µl of the sample was ultracentrifuged at 22 °C and 205,000 × *g* for 19 min (TLA 100.3, Beckman Coulter Inc., Fullerton, California). The experiments were performed in HEPES buffer. After separating the supernatant from the pellet both were used for SDS-PAGE analysis.

#### LUV uniformity analysis by DLS

The LUV suspension (with and without protein) was diluted to 0.2 mg/ml and set into the measurement chamber of a Zetasizer Nano-S (Malvern Instruments Ltd., Worcestershire, UK). The data was acquired by three independent measurements, each one consisting of 21 repetitions at 25 °C and using the Zetasizer Software 7.01 (Malvern Instruments Ltd., Worcestershire, UK).

#### FRET assay

FRET measurements were performed to detect the oligomerization of ENTH domains and the interaction between these proteins and specific lipids. Atto488 labeled ENTH domains were used as donor and Atto532 coupled to DOPS, DOPE (ATTO-TEC GmbH, Siegen, Germany), or ENTH domains were used as acceptor. The donor was excited at 493 nm and the acceptor emission maximum is at 552 nm. A total concentration of 5 and 15 µM labeled ENTH domain added into a 2 mg/ml lipid suspension. The FRET experiments were performed in a F-7000 Fluorescence Spectrophotometer (Hitachi K. K., Tokio, Japan) and the emission spectra were recorded between 500 and 700 nm.

#### Fluorescence labeling of ENTH domains

The ENTH WT (C96A A155C) domain and the ENTH R114A (C96A A155C) were labeled with Atto488 maleimide (or Atto532 maleimide) (ATTO-TEC GmbH, Siegen, Germany) by cysteine modification. The substitution reaction took place in PBS (136 mM NaCl, 2.7 mM KCl, 8 mM Na_2_HPO_4_, 1.8 mM K_2_HPO_4_, pH 7.4) with a molar access of the fluorophore of 1.4 in respect to the protein. The substitution was performed at 4 °C with gentle agitation on a tumbling shaker for 16 h.

Proteins were separated from free fluorophores by size-exclusion chromatography with a PD MiniTrap G-25 prepacked columns (GE Healthcare Life Science, Chalfont St Giles, UK). Finally, the degree of labeling (DoL) of the proteins was determined spectrophotometrically by measuring the UV–Vis (NanoDrop 2000, Thermo Fisher Scientific Inc., Waltham, USA). For this purpose, the following equation was used:

$${\text{DoL }} = A_{{\max}} \times \varepsilon_{{{\text{prot}}}} \times (A_{{{28}0}} - \, (A_{{\max}} \times {\text{CF}}_{{{28}0}} )^{{ - {1}}} \times \varepsilon_{{\max}}^{{ - {1}}} .$$

*A*_max_ is the absorption at the characteristic wavelength of the fluorophore, *ε*_max_ is the molar extinction coefficient of the fluorophore, A_280_ is the absorption of the protein at 280 nm, *ε*_prot_ is the molar extinction coefficient of the labeled protein and CF_280_ is the correction factor of the fluorophore.

### RIfS and AFM

#### Vesicle preparation

1-Palmitoyl-2-oleoyl-*sn*-glycero-3-phosphocholine (POPC), 1-palmitoyl-2-oleoyl-*sn*-glycero-3-phospho-L-serine (POPS), 1,2-dioleoyl-*sn*-glycero-3-phosphocholine (DOPC), 1,2-dioleoyl-*sn*-glycero-3-phosphoethanolamine (DOPE), 1,2-dioleoyl-*sn*-glycero-3-phospho-L-serine (DOPS) and L-α-phosphatidylinositol-4,5-bisphosphate (PIP_2_) were obtained from Avanti Polar Lipids (Alabaster, AL). Sulforhodamine-1,2-dihexanoyl-*sn*-glycero-3-phosphoethanolamine (Texas Red DHPE) was purchased from Sigma-Aldrich (Taufkirchen, Germany) and silicon wafers from Silicon Materials (Kaufering, Germany).

Stock solutions of lipids were prepared in chloroform at a concentration of 2–10 mg/mL. For PI(4,5)P_2_ a mixture of chloroform/methanol was used. The lipid stock solutions and fluorophores were added to a test tube filled with 400 µL chloroform and 30 µL methanol, obtaining the desired molar ratio. After removing the solvent in a nitrogen flush the lipid film was further dried under vacuum at 30° C for 3 h and stored at 4° C until use.

To obtain small unilamellar vesicles (SUVs), the lipid films (0.4 mg) were rehydrated with 0.5 mL citrate buffer (20 mM Na-citrate, 50 mM KCl, 0.1 mM EDTA, 0.1 mM NaN_3_, pH 4.8) for 30 min, followed by vortexing (3 × 30 s in a 5 min interval). By sonication of the lipid suspension for 30 min (60% power, Sonopuls HD2070, Bandelin; Berlin, Germany) the SUV suspension was gained.

#### Preparation of solid supported lipid membranes

For the AFM and RIfS experiments supported lipid membranes (SLBs) were prepared on silicon dioxide. Silicon wafers with 100 nm SiO_2_ (AFM) and 5000 nm SiO_2_ (RIfS) were cut into 0.8 × 1.9 cm^2^ rectangular pieces and treated in an aqueous solution of NH_3_ and H_2_O_2_ (H_2_O/NH_3_/H_2_O_2_ 5:1:1) for 30 min at 70 °C to obtain a hydrophilic surface. Further hydrophilization was reached by oxygen plasma treatment (30 s, 60% power).

The AFM substrates were fixed in a Teflon chamber and incubated with SUVs consisting of DOPC/DOPE/PI(4,5)P_2_/Texas Red DHPE (64.9:30:5:0.1) and DOPC/DOPE/DOPS/PI(4,5)P_2_/Texas Red DHPE (44.9:30:20:5:0.1) for 60 min. Rinsing with citrate buffer and PBS (1.5 mM KH_2_PO_4_, 8.1 mM Na_2_HPO_4_, 2.7 mM KCl, 136.9 mM NaCl, pH 7.4) removed remaining lipid material. The time-resolved SLB formation was monitored with RIfS.

#### Reflectometric interference spectroscopy (RIfS) 

The formation of SLBs and the protein adsorption were monitored using the fiber optic spectrometer SD2000 (Ocean Optics, Dunedin, USA) and the miniature spectrometer flame (Ocean Optics, Dunedin, USA). The experimental setup has been described previously [15]. The recorded spectra (every 2 s) were evaluated with a MATLAB routine. The hydrophilized substrates were mounted in a measuring cell and rinsed with ultrapure water and citrate buffer. SUVs of desired lipid compositions were added (circulating conditions). After reaching a constant change in optical thickness, indicating successful spreading of a bilayer, the substrates were rinsed with PBS to remove remaining lipid material. Passivation with BSA solution (1 mg/mL in PBS) prevented non-specific interactions. Afterwards the system again was rinsed with PBS. Then 0.1–5 µM ENTH^WT^ and ENTH^R114A^ were added until a plateau was reached. Rinsing with PBS resulted in ENTH desorption.

#### Atomic force microscopy (AFM) 

To investigate the protein coverage and protein height, atomic force micrographs of the SLB surface before and after protein addition were taken. ENTH^WT^ and ENTH^R114A^ (1 µM) were added for 2 h at RT. The solution was mixed with a stirring bar to ensure homogenous distribution of the protein. Afterwards 10 × 10 µm^2^ and 1 × 1 µm^2^ areas of the substrates were imaged in contact mode with BL-AC40TS-C2 cantilevers (*f* = 85.4–139.1 kHz, *k* = 0.03–0.12 N m^−1^, Olympus, Tokio, Japan). Measurements were performed using a JPK Nanowizard 4 (JPK Instruments, Berlin, Germany) equipped with a CCD camera (Fire Wire CCD color camera, The Imaging Source, NC, USA). The protein height and cluster surface coverage were calculated with a MATLAB routine.

### Cell biology

Plasmids: pEGFP-N1 (Clonetech), pEGFPC1-epsin 1 [16]. pEGFPC1-epsin 1^R114A^ was prepared by QuikChange II Site-directed mutagenesis kit (Agilent) using the pEGFPC1-epsin 1 as a template. Suppression (knock-down) of epsin 1, 2 and 3 expression in HeLa (ATCC® CCL-2™) cells by RNA interference approach was performed as described in [17]. This approach was taken due to the redundant functions of epsin 1, 2 and 3 [7]. Here, Lipofectamine RNAiMax (ThermoFisher) was used to introduce 200 nM scrambled or three different DsiRNAs (Integrated DNA Technologies, Inc. (IDT), Coralville, Iowa, Epsin1: hs.Ri.EPN1.13.3; Epsin2: hs.Ri.EPN2.13.1; Epsin3: hs.Ri.EPN3.13.3) into HeLa cells two times (with a 24 h interval) and plated on poly-l-lysine coated glass coverslips (Marienfeld, Cat No. 0117580; Ø18 mm) in DMEM medium containing DMEM (Gibco), 10% fetal bovine serum (FBS) and penicillin/streptomycin (Gibco). The efficiency of KD was inspected by quantitative PCR (qPCR; Figure S4A), as in Boucrot et al. [7], since the specific antibodies for three epsin proteins (1, 2 and 3) are not commercially available. After 20 h on coated coverslips, cells were transfected with either EGFP, epsin 1^WT^-EGFP or epsin 1^R114A^-EGFP using Fugene 6 transfection reagent (Promega). For the fluorescent uptake assays, 28–32 h after transfection cells were serum-starved for 1 h at 37 °C in pre-warmed DMEM containing 20 mM HEPES, pH 7.4, and 1% BSA, and then incubated with biotinylated Epidermal Growth Factor (EGF) complexed to Texas Red™ Streptavidin (ThermoFischer/Molecular Probes Cat. No. E3480; referred to as EGF-Texas Red in the text and figures; 2 ng/mL) or Alexa Fluor 548–transferrin (ThermoFischer/Molecular probes Cat. No. T23364; referred to as transferrin-A548 in the text and figures; 50 μg/mL) on ice for 30 min. Subsequently, cells were washed once in ice-cold PBS and then placed in pre-warmed DMEM media for indicated time at 37 °C. After incubation, cells were placed on ice and incubated in cold acidic PBS (pH 4.5) for 5 min on ice to remove fluorescent EGF or transferrin from the cell surface. The cells were then washed two times with ice-cold PBS (pH 7.4), fixed and processed for immunofluorescence.

In experiments with Pitstop-2 (Abcam, Cat. ab120687), 20 µM of the inhibitor was added 30 min before the experiment was started, and kept throughout the transferrin-A548 uptake. Knock-down of clathrin light chain (LC) expression in HeLa cells was performed using a mixture of 4 siRNA named ON-TARGETplus Human CLTC (Dharmacon): 100 nM SMARTpool during 48 h reduced clathrin LC expression at the mRNA level by ~ 78%. Negative control siRNA was also purchased from Dharmacon. Since multiple transfections were performed subsequently (Lipofectamine RNAiMax was used for SiRNAs, Fugene 6 was used for epsin 1^WT^-EGFP or epsin 1^R114A^-EGFP), HeLa cells attached to the glass coverslips were kept in Opti-MEM (Gibco) during transfections at 37 °C, and then transferred and kept in fresh DMEM medium at 37 °C.

Images were captured with a commercial confocal Zeiss 800 Airyscan microscope setup (Carl Zeiss Inc.). The acquired images were analyzed using Metamorph software 6.1. (Molecular Devices). Image background is subtracted from fluorescence intensity measurement of each cell.

For flow cytometry experiments, epsin 1,2,3-KD HeLa cells were plated after electroporation on poly-L-lysine-coated plastic petri dishes (Sarstadt Ø18 mm) in the DMEM media (Gibco) containing 10% fetal bovine serum (FBS) for 20–22 h, and transfected with either EGFP, epsin 1 WT-EGFP or epsin 1^R114A^-EGFP as before. 28–34 h after transfection, cells were serum-starved for 1 h at 37 °C in pre-warmed DMEM containing 20 mM HEPES, pH 7.4, and 1% bovine serum albumin (BSA) and then incubated with biotinylated Epidermal Growth Factor (EGF) complexed to Texas Red™ Streptavidin (ThermoFischer/Molecular Probes Cat. E3480; referred to as EGF-Texas Red in the text and figures; 2 ng/mL) or Alexa Fluor 548–transferrin (ThermoFischer/Molecular probes Cat. No. T23364; referred to as transferrin-A548 in the text and figures; 50 μg/mL) on ice for 30 min, washed once in ice-cold PBS and then placed in pre-warmed DMEM media for indicated time at 37 °C. After incubation, cells were placed on ice and incubated in cold acidic PBS (pH 4.5) for 5 min on ice to remove fluorescent EGF or transferrin from the cell surface. Cells were then harvested from the plates and processed for flow cytometry. Fluorescence-activated cell sorting and counting was done by Sony Cell Sorter SH800S. FACS data were analyzed by FlowJo software and plotted in Prism (GraphPad). Data are analyzed and displayed using Sigma Plot 12.5 (Systat Software, Inc.), Prism 7 (GraphPad) and Adobe Photoshop 10 (Adobe). Statistical significance was set at *p* < 0.05 using unpaired Student’s *t* test for two group comparisons (flow cytometry) and one-way ANOVA (uptake assays).

### NMR spectroscopy

NMR samples containing ENTH and its variant R119 were packed in a 3.2 mm rotor with DSS added externally for temperature calibration. Experiments were recorded on a 850 MHz wide-bore spectrometer equipped with 3.2 mm triple resonance probe (Bruker Biospin). All ssNMR experiments were recorded at 5 °C. 90° pulse widths of 3 μs for ^1^H, 5 μs for ^13^C, 7 μs for ^15^N and decoupling strength of ~ 70–80 kHz was used for CP and C90 experiment. Spectra were processed and analyzed in Topspin. The samples were spun at 8 kHz for INEPT experiment, 11 kHz for CP and C90 experiment.

## Results

### Epsin1 ENTH domain induces phosphatidylserine-dependent membrane curvature

**Fig. 1:**
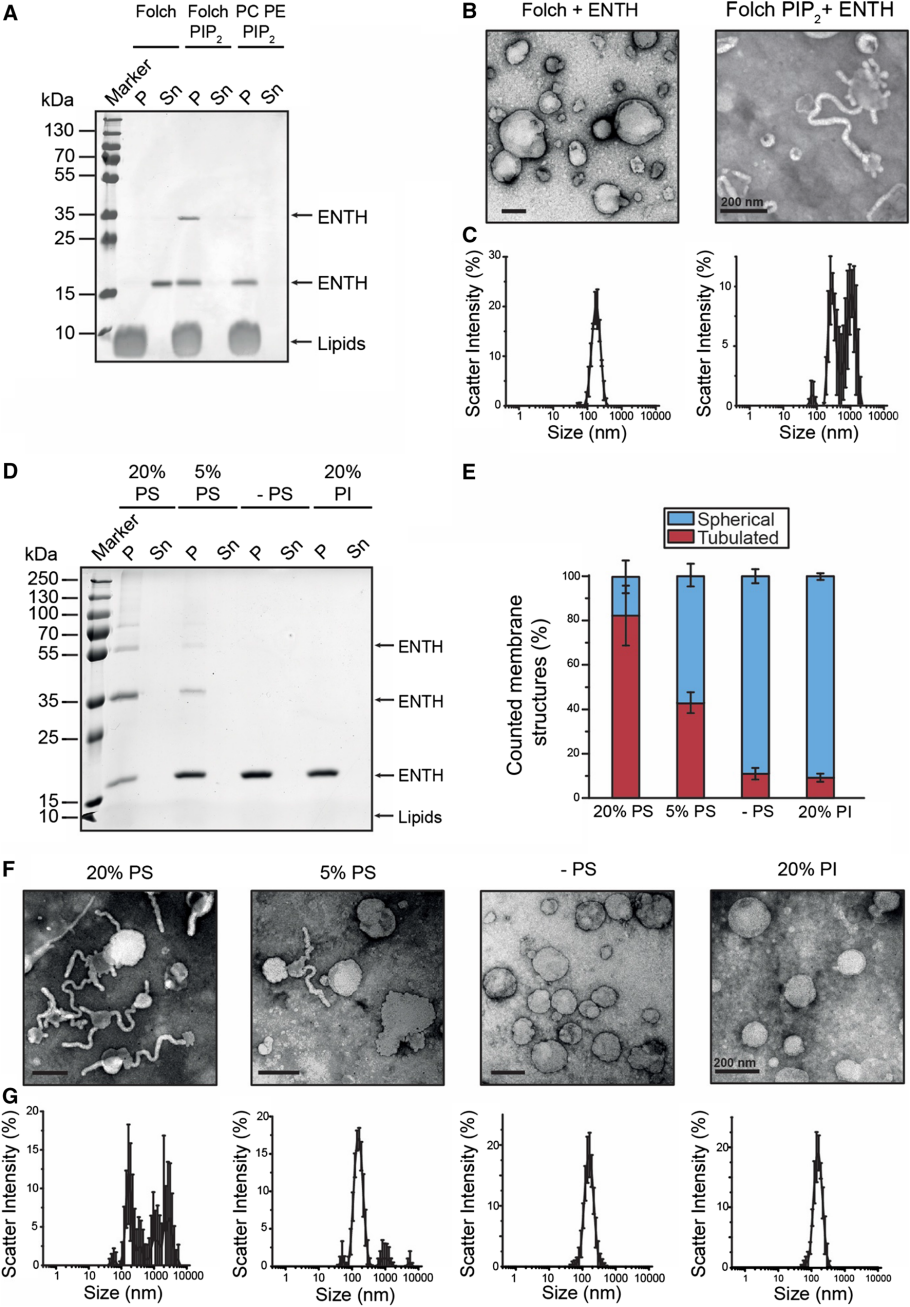
ENTH deforms membranes in a PS-dependent manner. **a** SDS-PAGE of co-sedimentation shows that ENTH domain binding to LUVs only depends on the presence PI(4,5)P_2_. LUVs consisted of polar brain lipid extract (Folch) in the absence and presence of PIP2 as indicated (Folch: PI(4,5)P_2_ (95: 5 mol%)). **b** ENTH domain induced membrane deformation upon LUV binding as analyzed by electron microscopy. Lipid composition as in (**a**). **c** Dynamic light scattering spectra of LUVs shown in (**b**) showed PIP2-dependent alterations DLS data were acquired by 3 times 3 independent measurements, each one consisting of 21 repetitions for each lipid composition. Error bars represent the standard deviation. **d** SDS-PAGE of co-sedimentation of ENTH domain with LUVs of indicated lipid composition LUVs containing PS were composed of PC, PE, PS and PI(4,5)P_2_ (20% PS—45: 30: 20: 5 mol%; 5% PS—60: 30: 5: 5 mol%). LUVs without PS were composed of PC, PE and PI(4,5)P_2_ (0% PS—65: 30: 5 mol%) and LUVs containing PI were composed of PC, PE, PI, PI(4,5)P_2_ (20% PI—45: 30: 20: 5 mol%). **e** Statistical evaluation of EM micrographs shown in (**f**). Error bars represent the standard error of the mean (SEM). **f **Electron microscopy of negatively stained LUVs with indicated increasing PS or PI concentrations. LUVs were composed with similar lipid compositions as in (**d**). At least 250 membrane structures were counted for each condition and the error bars represent the standard deviation. **g** Dynamic light scattering spectra of LUVs shown in (**e**). DLS data was acquired by 3 independent measurements, each one consisting of 21 repetitions for each lipid composition. Each measuring circle was repeated 3 times

The role of the membrane lipid composition in the process of protein-induced membrane deformation is poorly understood. Due to the complexity and heterogeneity of biological membranes in vitro studies usually employ ill-defined lipid mixtures derived from native tissue or very simple compositions that show little resemblance to the in vivo situation. Often these two different approaches lead to results that are difficult to compare, or are even contradicting. When analyzing the well-studied curvature-inducing activity of epsin1 ENTH domain, we observed that the ENTH domain is able to bind to large unilamellar vesicles (LUVs) of different membrane composition in a PI(4,5)P_2_-dependent manner, as described before (Fig. 1a) [8, 18]. Surprisingly though, we found that while addition of the ENTH domain to vesicles made from brain-derived lipid mixtures led to deformed membranes (Fig. 1b, c), no signs of membrane remodeling was observed in liposomes formed from phosphatidylcholine (PC), phosphatidylethanolamine (PE) and PI(4,5)P_2_ (Fig. 1f (-PS)). These effects could be visualized by electron microscopy (Fig. 1b, f) and in bulk by analyzing the size distribution of LUVs by dynamic light scattering (Fig. 1c, g).

PS is the most abundant anionic cellular phospholipid. In resting cells, it is with at least 15% exclusively found in the inner leaflet of the plasma membrane [19]. It is also part of the brained-derived lipid composition. We hence asked if the presence of PS would influence membrane deformation by the ENTH domain. The ENTH domain of epsin1 efficiently bound to LUVs consisting of PC, PE, PI(4,5)P_2_ and increasing amounts of PS (Fig. 1d). Membrane deformation was dependent on the presence of PS and statistical evaluation of electron micrographs of LUVs revealed a clear correlation between PS concentration and membrane remodeling (Fig. 1e, f). Similar results were obtained using giant unilamellar vesicles (GUVs) (Figure S1A–B). Membrane curvature induction was specific to PS as the presence of another anionic lipid, phosphatidylinositol (PI), did not lead to deformed membranes (Fig. 1e–g). High concentrations of unsaturated PE also did not lead to membrane deformation, ruling out that changes of membrane morphology are induced by high concentration of non-bilayer lipids (Figure S1C).

### PS induces ENTH domain oligomerization

**Fig. 2:**
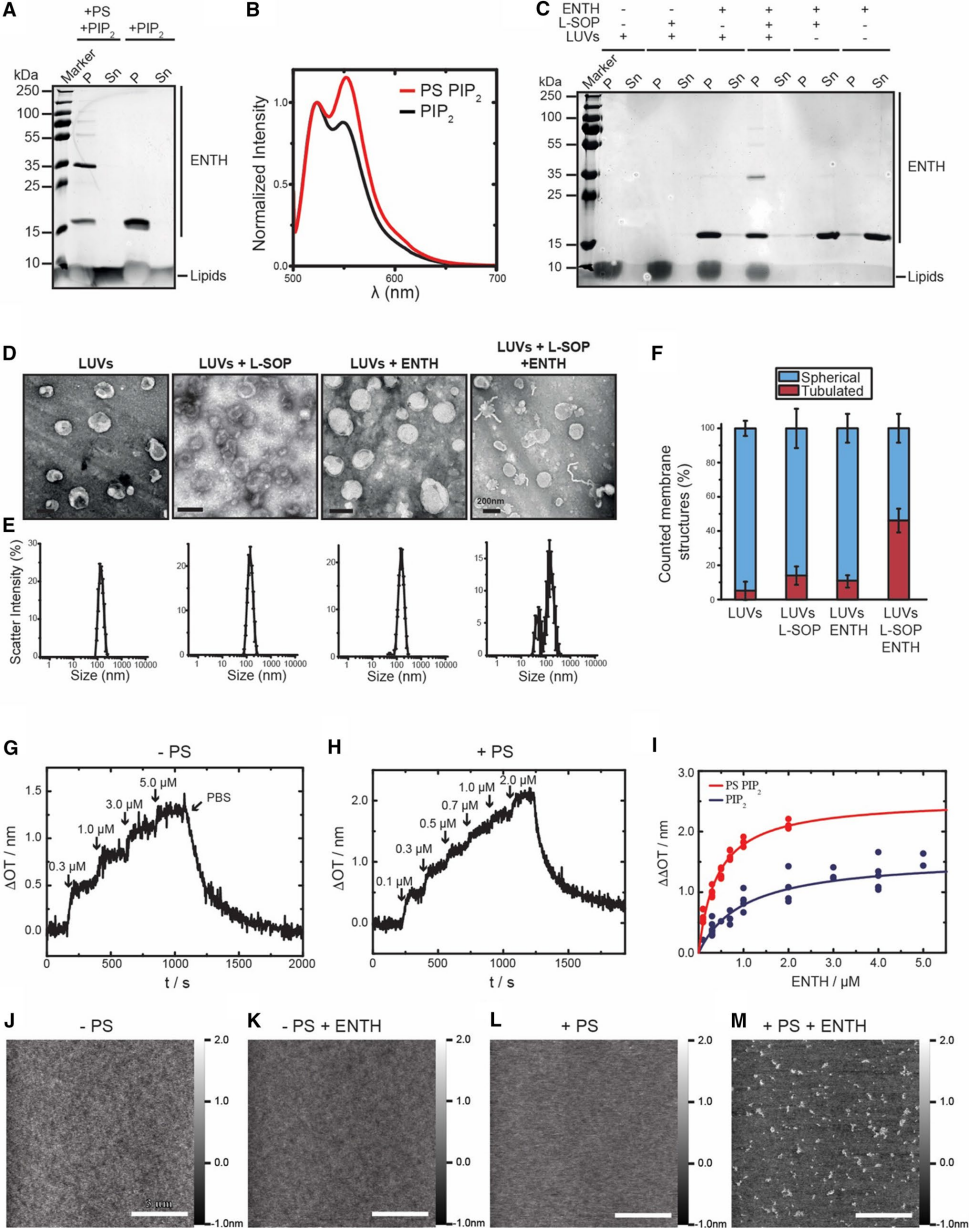
PS-dependent oligomerization of ENTH. **a** SDS-PAGE of a co-sedimentation spin assay of the ENTH domain was accompanied by the formation of homo-oligomers in presence of PS. LUVs were composed of PC, PE, PS and PI(4,5)P_2_ (PS PIP_2_—45: 30: 20: 5 mol%) or PC, PE and PI(4,5)P_2_ (PIP_2_—65: 30: 5 mol%). **b** Protein–protein interaction is demonstrated by FRET assay in which the peak-maximum normalized spectra are shown. For FRET experiments two differently labeled ENTH domain populations were generated, the one was labeled with Atto488 maleimide, the other population was labeled with Atto532 maleimide. LUVs were composed as described in (**a**). **c** SDS-PAGE of ENTH domain co-sedimentation assay in the absence and presence of LUVs or PS headgroups, as indicated. L-SOP was used in a 4 times molar excess to the ENTH domain. LUVs were composed of PC, PE and PI(4,5)P_2_ (PIP_2_—65: 30: 5 mol%). **d** Vesicle deformation assay analyzed by electron microscopy of LUVS, under indicated conditions shows that L-SOP is able to partially recue membrane deformation on LUVs. L-SOP was used in a 4 times molar excess to the ENTH domain and LUVs were composed of PC, PE and PI(4,5)P_2_ (PIP_2_—65: 30: 5 mol%). Scale bars correspond to 200 nm. **e** Dynamic light scattering spectra of LUVs shown in (**d**). DLS data were acquired by 3 times 3 independent measurements, each one consisting of 21 repetitions for each lipid composition. Error bars represent the standard error of the mean (SEM). **f** Statistical evaluation of the micrographs displayed in (**d**) At least 150 membrane structures were counted. The error bars were calculated by the standard deviation. **g** Adsorption of ENTH^WT^ to a POPC/PI(4,5)P_2_ (95:5) bilayer. The stepwise increase in Δ*OT* upon addition of different ENTH concentrations (marked by arrows) shows the specific binding of ENTH to PI(4,5)P_2_. Rinsing with PBS results in desorption of the protein indicating reversible binding. **h** Time-resolved change in optical thickness upon addition of different ENTH^WT^ concentrations (marked by arrows) to a POPC/POPS/PI(4,5)P_2_ (75:20:5) bilayer. After rinsing with PBS, almost all bound protein desorbs from the membrane indicating reversible binding. **i** Adsorption isotherms of ENTH^WT^ to POPC/PI(4,5)P_2_ (95:5) (blue circles) and POPC/POPS/PI(4,5)P_2_ (75:20:5) (red circles) bilayers. A Langmuir adsorption isotherm was fitted to the data (solid lines). Non-linear regression weighted by the corresponding number of measurements that went into each concentration was carried out using a Levenberg–Marquardt algorithm. The obtained values for ΔΔ*OT*_max_ and *K*_D_ are summarized in Table 1. **J–m** Atomic force micrographs of (**j**) DOPC/DOPE/PI(4,5)P_2_/Texas Red DHPE (64.9/30/5/0.1) and (**l**) DOPC/DOPE/DOPS/PI(4,5)P_2_/Texas Red DHPE (44.9/30/20/0.1) bilayers on hydrophilic silicon dioxide wafers prior ENTH addition. **k** and **m** show the corresponding micrographs after 2 h of ENTH^WT^ incubation (1 µM). Only in the presence of DOPS, protein clusters were observed on the membrane surface. From the topography images, the occupancy of 6 ± 1% and the protein height of 1.2 ± 0.2 nm (values ± SD, *n* = 32, with *n* the number of evaluated images from three independent experiments) were calculated

The liposome co-sedimentation assays showed that the ENTH domain of epsin1 binds to membranes in a PI(4,5)P_2_-dependent manner, as published before (Fig. 1a). The amount of bound protein did not vary with changing lipid compositions (Fig. 1a, d). However, when analyzing the fractions of liposome bound proteins, SDS resistant oligomers were detected when using Folch liposomes (Fig. 1a). The same tendency for oligomerization was observed with increasing PS concentration (Figs. 1d, 2a). As the appearance of PS-dependent oligomers coincided with membrane deformation, we analyzed this effect in more detail. We first tested if oligomerization not only occurs in SDS resolved samples but can also take place on membranes. We therefore performed FRET (Förster resonance energy transfer) experiments of ENTH domain in the presence of PS-free and PS-containing LUVs. Efficient FRET signals were detected when two different fractions of fluorescently labeled protein (ENTH-Atto-488 [donor] and ENTH-Atto-532 [acceptor]) were incubated with PS-containing liposomes (Fig. 2b). PS-free LUVs led to reduced FRET signals, confirming that ENTH molecules are in close proximity on PS-containing membranes. Interestingly, when repeating membrane binding and membrane deformation with PI(4,5)P_2_-containing liposomes and ENTH domains, we observed that the presence of the PS headgroup was sufficient to partially restore oligomerization and membrane remodeling (Fig. 2c–f).

**Table 1:** Summary of the fit results of the Langmuir adsorption isotherms for ENTH^WT^ and ENTH^R114A^

	*K*_D_		ΔΔOT_max_	
	ENTH^WT^	ENTH^R114A^	ENTH^WT^	ENTH^R114A^
No PS	1.0 ± 0.2 µM	2.9 ± 0.8 µM	1.6 ± 0.1 nm	1.5 ± 0.2 nm
with PS	0.42 ± 0.05 µM	1.0 ± 0.3 µM	2.5 ± 0.1 nm	2.7 ± 0.2 nm

The values are given as parameter ± SE

To understand the molecular details of this lipid-specific protein-membrane interaction, we next quantitatively investigated the behavior of the ENTH domain on different membranes. No signs of ENTH domain membrane binding were observed in the absence of PI(4,5)P_2_ by reflectometric interference spectroscopy (RIfS) (Figure S2B). When the ENTH domain was added to supported bilayers containing PI(4,5)P_2_ an increase in optical thickness was observed (Fig. 2g). When PS was included in these experiments, the increase was larger (Fig. 2h). Plotting the change in optical thickness against the ENTH domain concentrations allowed us to calculate binding affinities for membranes of different composition. The dissociation constant *K*_D_ for PI(4,5)P_2_-containing PC membranes was about 1 µM. *K*_D_ decreased to about 0.4 µM when the membranes contained 20% PS (Fig. 2i), indicating a higher binding affinity of ENTH to PS-containing membranes. We then sought to analyze structural details of the PS-dependent oligomer formation and higher protein affinity. When the ENTH domain was added to PI(4,5)P_2_-containing bilayers in the absence/presence of PS and the membrane surface was analyzed by atomic force microscopy (AFM) clear protein clusters could be observed in a PS-dependent manner (Fig. 2k–m). Whereas membranes of various composition in the absence of proteins showed a rather smooth surface, the addition of ENTH domain led to protein clusters or oligomers, with a height of about 1.2 nm (Figure S2C), in line with the biochemical and FRET results. Control experiments with PS-free membranes, in which no signs of protein clusters were observed (Fig. 2k), support the idea of PS-dependent ENTH oligomerization.

To further analyze the structural differences of membrane bound ENTH domains in the absence or presence of PS, we used solid-state NMR spectroscopy. The ENTH domain was ^13^C/^15^N-labeled by expressing the protein in *E. coli* cells grown in buffer supplemented with ^15^N ammonium chloride and ^13^C glucose. Though the measured solid-state NMR spectra were too heterogeneous to reach atomic resolution, comparison of 1D ^13^C-spectra showed clear differences between the presence of PS, where ENTH is able to tubulate membranes, and the absence of PS (Figure S2D–G). ^1^H–^13^C CP experiments revealed an increased signal intensity in C^α^ and C^β^ regions in the presence of PS (Figure S2F), which is an indicator of increased rigidity of the protein. A rigid protein population with a reduced number of motions supports the results showing PS-dependent oligomerization in biochemical, FRET and AFM experiments. It also explains results from AFM measurements in the absence of PS. The relatively even surface observed in these experiments is indicative of mobile protein monomers that cannot be resolved by AFM (Fig. 2k).

### A single amino acid exchange abolishes ENTH domain oligomerization and membrane deformation

**Fig. 3:**
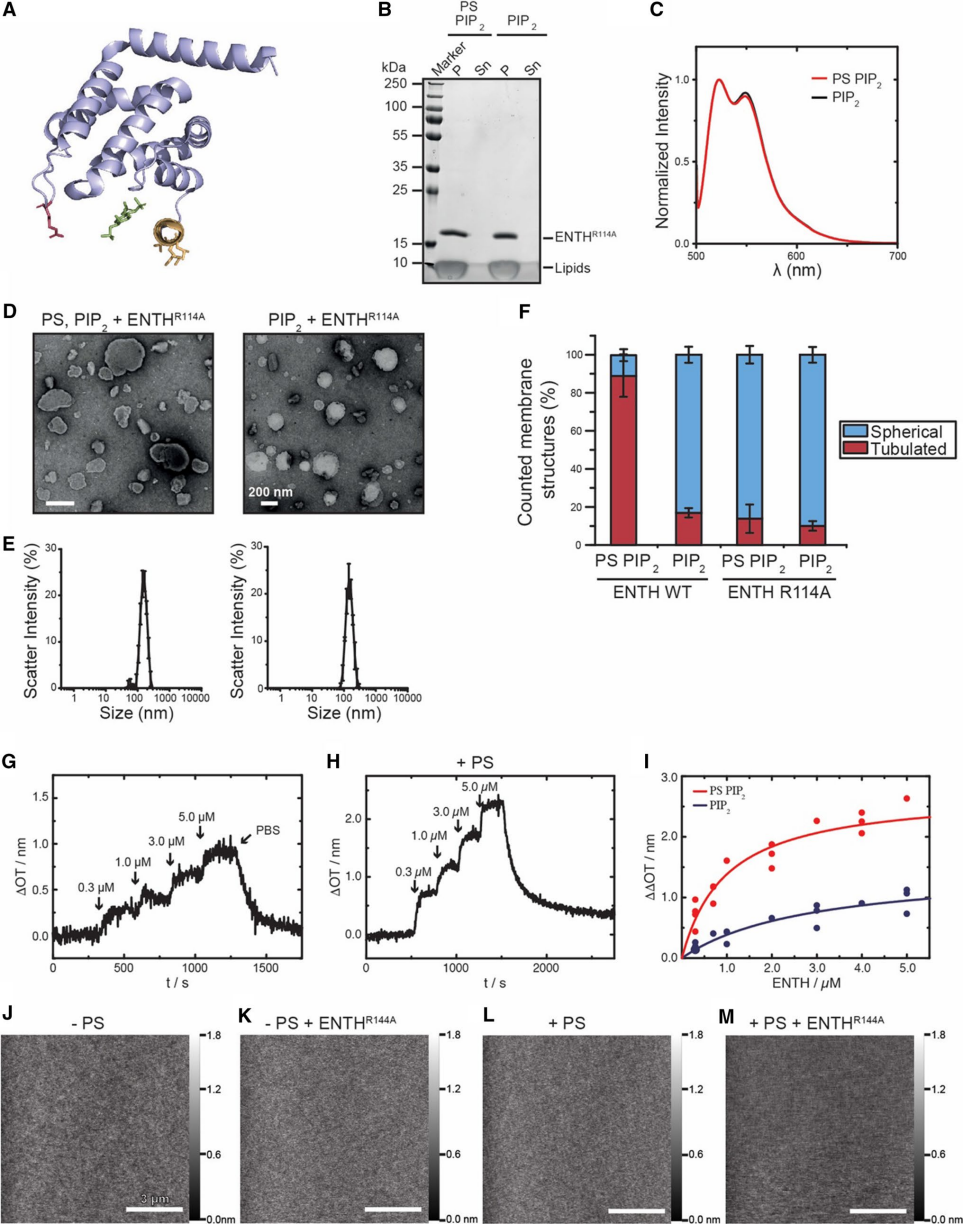
A point mutation in ENTH eliminates PS-dependent oligomerization and curvature induction. **a** Arginine 114 (R114) is located within the loop l6,7 in the crystal structure of the ENTH domain. The amphipathic helix (yellow) structures upon interaction with the PI(4,5)P_2_ headgroup inositol (1,4,5)-triphosphate (green). This arginine was mutated to alanine by site-directed mutagenesis (ENTH^R114A^). **b** SDS-PAGE of co-sedimentation assay of ENTH^R114A^ with LUVs. LUVs used for this spin assay were composed of PC, PE, PS, PI(4,5)P_2_ (“PS PIP_2_”—PC 45: 30: 20: 5 mol%; “PIP_2_”—65: 30: 5 mol%). **c** ENTH^R114A^ protein–protein interaction as analyzed by FRET. Two differently labeled protein populations were generated, one labeled with Atto532, the other one labeled with Atto488. LUVs for the experiment were composed of PC, PE, PS and PI(4,5)P_2_: (“PS PIP_2_”—45: 30: 20: 5)mol%; PIP_2_—65: 30: 5 mol%). **d** Membrane deformation analyzed by electron microscopy of negatively stained LUVs under indicated conditions. LUVs were composed as described before (see **b**) and scale bars correspond to 200 nm. **e** Dynamic light scattering spectra of LUVs shown in (**d**). DLS data were acquired by 3 times 3 independent measurements, each one consisting of 21 repetitions for each lipid composition. Error bars represent the standard error of the mean (SEM). **f** Statistical evaluation of the micrographs displayed in (**d**) At least 150 membrane structures were counted. The error bars were calculated by the standard deviation. **g** Adsorption of ENTH^R114A^ to a POPC/PI(4,5)P_2_ (95:5) bilayer. Upon addition of different ENTH^R114A^ concentrations (marked by arrows) a stepwise increase in Δ*OT* occurs showing the specific binding of ENTH^R114A^ to PI(4,5)P_2_. After rinsing with PBS, the protein desorbs from the membrane indicating reversible binding. **h** Time-resolved change in optical thickness upon addition of different ENTH^R114A^ concentrations (marked by arrows) to a POPC/POPS/PI(4,5)P_2_ (75:20:5) bilayer. The protein desorbs after rinsing with PBS showing the reversibility of binding. **i** Adsorption isotherms of ENTH^R114A^ to POPC/PI(4,5)P_2_ (95:5) (blue circles) and POPC/POPS/PI(4,5)P_2_ (75:20:5) (red circles) bilayers. The values for ΔΔ*OT*_max_ and *K*_D_ (Table 1) were obtained by fitting a Langmuir adsorption isotherm (solid lines) to the data. Non-linear regression weighted by the corresponding number of measurements that went into each concentration was carried out using a Levenberg–Marquardt algorithm. **j–m** Atomic force micrographs of (**k**) DOPC/DOPE/PI(4,5)P_2_/Texas Red DHPE (64.9/30/5/0.1) and (**m**) DOPC/DOPE/DOPS/PI(4,5)P_2_/Texas Red DHPE (44.9/30/20/0.1) bilayers on hydrophilic silicon dioxide wafers prior ENTH addition. The corresponding micrographs (**l**, **n**) were obtained after 2 h of ENTH^R114A^ incubation (1 µM). Even in the presence of DOPS, no protein clusters were observed on the membrane surface indicating the necessity of the amino acid R114 for ENTH cluster formation

It is well-studied that upon binding to PI(4,5)P_2_ an unstructured N-terminal stretch of the ENTH domain folds into an amphipathic α-helix that inserts into the membrane [20, 21]. It is however not obvious how this mode of binding allows the observed PS-dependency. In models of the membrane bound ENTH domain based on EPR measurements a self-assembly was suggested, and beside the hydrophobic PI(4,5)P_2_ binding pocket and the amphipathic helix 0 a loop region was found in close proximity to the membrane [20, 22]. Interestingly, an arginine residue at position 114 is located at the tip of this loop (Fig. 3a).

Having found a regulatory effect of the anionic phospholipid PS we next asked the question if membrane binding and/or membrane remodeling of the ENTH could be influenced by this amino acid residue. ENTH domains with a single amino acid exchange from arginine to alanine (R114A) were constructed and analyzed in a similar fashion as the wild-type (WT) protein. The mutation did not affect the overall structure of the ENTH domain as secondary structures of WT and mutant proteins showed no differences when measured by circular dichroism (CD) spectroscopy (Figure S3A and S3B). Membrane binding was again observed in a strictly PI(4,5)P_2_-dependent manner. Importantly though, even increasing amounts of PS in LUVs did not lead to SDS resistant oligomers that were observed with ENTH^WT^ (Fig. 3B; Figure S3C). Analysis of differentially labeled membrane bound ENTH^R114A^ showed no PS-dependent differences in FRET efficiency underlining that the mutant fails to form homo-oligomers (Fig. 3c). When the same vesicles in absence and presence of PS were incubated with ENTH^R114A^ and analyzed for membrane deformation by electron microscopy and dynamic light scattering, no signs of membrane remodeling were observed despite high concentrations of PS (Fig. 3d–f). Similarly, no membrane curvature induction was observed when GUVs were incubated with ENTH^R114A^ (Figure S3D). RIfS measurements with ENTH^R114A^ showed that the mutant is able to bind to membranes in the absence and presence of PS, though with lower affinities than the wild-type protein (Fig. 3g–i). Nonetheless, when analyzed by AFM (Fig. 3j–m) the mutant protein exhibited no sign of PS-dependent cluster formation as the wild-type protein did (Fig. 3m). When ENTH^R114A^ was analyzed by solid-state NMR spectroscopy no differences in the spectra were detected between the protein bound to a PS-free or PS-containing membrane (Figure S3E-H). Taken together, these results clearly demonstrate that PS-induced oligomerization leads to membrane deformation, which is inhibited by a point mutation of the ENTH domain.

### Oligomer-dependent membrane deformation is important for clathrin-mediated endocytosis

**Fig. 4:**
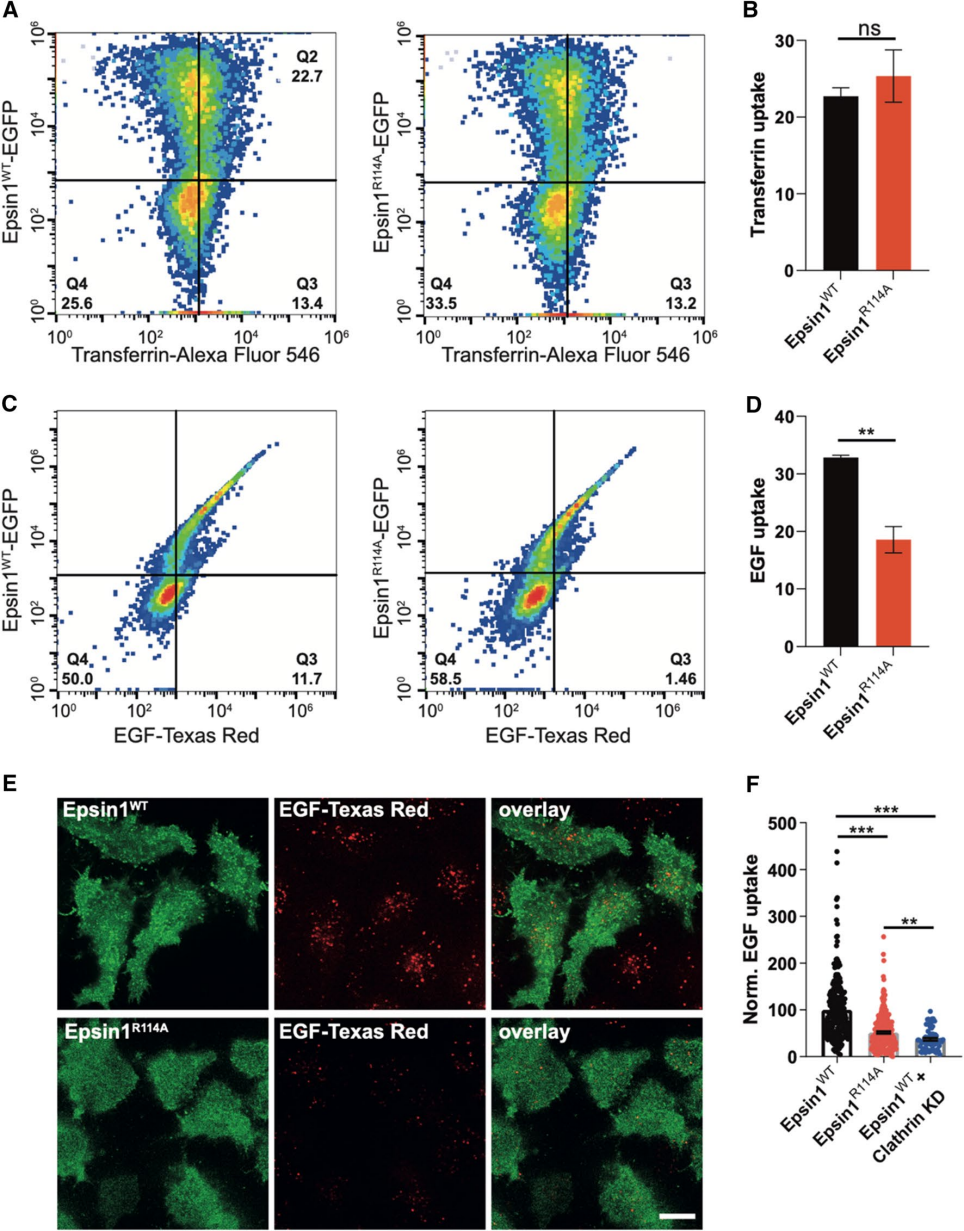
Epsin 1^R114A^ affects EGF uptake, but not transferrin uptake, by HeLa cells. **a**, **b** Epsin 1,2,3 KD HeLa cells expressing either epsin1^WT^-EGFP and epsin1^R114A^-EGFP were allowed to internalize transferrin-Alexa Fluor 548 and were subsequently analyzed by flow cytometry. Experiments were performed as detailed in Methods. 20,000 cells were counted per condition and experiment; four independent experiments were performed. mean ± SEM; ns = not significant (unpaired *t* test). **c**, **d** Epsin 1,2,3 KD HeLa cells expressing either epsin1^WT^-EGFP and epsin1^R114A^-EGFP were allowed to internalize EGF-Texas Red and were subsequently analyzed by flow cytometry. Experiments were performed as detailed in Methods. 20,000 cells were counted per condition and experiment; four independent experiments were performed. mean ± SEM; ∗*∗ p* = 0.007 (unpaired *t* test). **e** Representative images of epsin 1,2,3, KD HeLa cells expressing epsin1^WT^-EGFP and epsin1^R114A^-EGFP after EGF-Texas Red uptake. Optical sections through cells in the proximity of plasma membrane (footprints) were shown. Although not further analyzed, note that non-transfected (non-fluorescent in green channel) cells in both conditions showed slightly higher levels of internalized EGF-Texas Red. **f** Analysis of fluorescent EGF-Texas Red signal in epsin1^WT^-EGFP (black) or epsin1^R114A^-EGFP (red) cells. Data are presented as mean ± SEM. The number of analyzed cells: 237 for epsin1^WT^ and epsin1^R114A^ (4 experiments) and 64 for epsin1^WT^ with clathrin LC knock-down (2 experiments); ∗∗*p* < 0.05; ∗∗∗*p* < 0.001 (one-way ANOVA)

As we identified a mutant with a clear phenotype in in vitro investigations, we next asked if oligomer-dependent membrane remodeling is of physiological importance in clathrin-mediated endocytosis. To this end we performed fluorescent transferrin (Tf) and Epidermal Growth Factor (EGF) uptake assays in HeLa cells that were shown before to depend on epsin1 [6, 7]. Specifically, simultaneous depletion of epsin1, 2, and 3 resulted in a significant decrease in Tf uptake (Figure S4A-B), in agreement with Boucrot et al. [7]. This perturbation was shown to be specific to CME [7], which was also confirmed in our experiments, where inhibition of CME by knock-down of clathrin light chain or the clathrin inhibitor Pitstop-2 resulted in a similar decrease in Tf uptake as knock-down (KD) of epsin 1, 2 and 3 (Figure S4B). Expression of either epsin1^WT^-EGFP and epsin1^R114A^-EGFP in epsin 1,2,3, KD HeLa cells was able to restore Tf-A548 uptake to similar levels, as seen by flow cytometry (Fig. 4a, b). When EGF-Texas Red uptake was examined a different result was obtained: epsin 1,2,3, KD HeLa cells expressing epsin1^R114A^-EGFP were only able to internalize less fluorescent EGF than cells expressing epsin1^WT^ -EGFP under the same conditions (Fig. 4c, d). A similar result was observed when EGF-Texas Red uptake by epsin 1,2,3 KD HeLa-expressing epsin1^WT^-EGFP and epsin1^R114A^ -EGFP, respectively, was analyzed by fluorescence imaging (Fig. 4e, f). Notably, epsin 1,2,3 KD HeLa cells expressing epsin1^R114A^-EGFP internalized on average more EGF-Texas Red than epsin 1,2,3 KD HeLa cells where epsin1^WT^-EGFP is expressed and clathrin was knocked down (Fig. 4f), showing that epsin1^R114A^ interferes with, but does not abolish CME. Our uptake experiments also revealed that epsin1^R114A^ alters EGF uptake, but not Tf uptake.

## Discussion

We herein present the identification of a lipid-dependency for protein-induced membrane remodeling during CME for the ENTH domain of epsin1. We show that PS has a regulatory effect on epsin1 by combining in vitro reconstitution and quantitative biophysical analysis with cell biological approaches. The protein oligomerizes and induces changes in membrane morphology in a PS-dependent manner. Anionic lipids, especially PS, were implicated to be important for CME [23–26]. Increased vesicle endocytosis was found to be dependent on increased PS concentrations at the plasma membrane [25]. Additionally, it was shown that PS is involved in vesicle internalization, where the lipid stimulates fission rates [23]. While it is discussed that clustering of anionic phospholipids has a direct effect on membrane morphology [26], our results show a protein-lipid cooperativity and are, to the best of our knowledge, the first molecular explanation for the phenomenon that PS stimulates CME. They also touch on a neglected topic in membrane trafficking—the regulatory involvement of the membrane lipid composition on these processes. As noted before, many proteins operating in CME do not act separately but show cooperative behavior that is most probably necessary for the spatial and temporal fidelity of CME [27–30]. We now show that the action of proteins during CME is not only fine-tuned by mere membrane recruitment and regulatory protein–protein interactions, but that the membrane lipid composition also plays a crucial role in the regulation of protein-dependent membrane remodeling. This is all the more important as it is still poorly understood why particular proteins act on specific membranes, while being in principle able to bind to other cellular membranes, too. Due to the immense variety and dynamics of cellular lipids, a detailed molecular characterization of the regulatory effect of the membrane composition on membrane-based processes is still missing to a large degree. Nonetheless, sporadic reports are emerging and the importance of such interactions will be in all likelihood widespread. A recent paper showed a regulatory role of the membrane lipid composition, specifically membrane-charge, on the remodeling activity of the BAR domain-containing protein BIN1 [31]. Our in vitro findings clearly show that membrane bending by the ENTH domain does not rely on electrostatic interactions between PS and ENTH but suggest a more specific effect. PS headgroup binding to the ENTH domain induces oligomerization and membrane deformation, while other negatively charged lipids did not mimic this effect. These results also suggest that, at least in this case, the composition of the acyl chains is not a major contributor to ENTH-dependent membrane deformation. As it is well-known that negatively charged lipids have a tendency to cluster into microdomains [32–35], the net charge of PS could play a role in establishing PS-enriched membrane patches that, in turn, promote epsin oligomerization. Here, also the fatty acid chain composition becomes a factor as length and saturation is also known to play a part in membrane micro-domain formation [36].

CME is a process that depends on the spatial and temporal precise action of a complex set of different proteins [37–39]. These include not only clathrin itself, but also adaptor proteins. Together these proteins mediate cargo sorting, nucleation of the clathrin-coated pit, membrane remodeling during pit maturation and fission as well as release of the clathrin-coated vesicle. Since it is known that EGF-receptor endocytosis is minimally impacted by epsin1 single knock-out cells and even in epsin1 and 2 double knock-out cells [10, 40], it is intriguing that the expression of epsin1^R114A^ interferes with CME in a significant way. Here, epsin1^R114A^ may serve as a dominant negative effector by trapping ubiquitinated EGF receptors in membrane domains that fail to undergo necessary remodeling, due to the lack of oligomerization of the espin1^R114A^-ENTH domain. Transferrin-stimulated endocytosis, however, may occur at different membrane domains that undergo normal remodeling independently of epsins. Our uptake experiments also revealed that epsin1^R114A^ alters EGF uptake, but not Tf uptake. This is in agreement with reports that EGF-receptor internalization by CME is indeed dependent on epsins, especially in the absence of the adaptor complex AP-2 [41].

Besides being heavily studied for its physiological importance for cell signaling, nutrient uptake, migration or neurotransmission, to name just a few, CME has become a model system to study membrane remodeling proteins [1]. The vast majority of these proteins are peripheral, temporarily attached membrane proteins [3, 39]. They can be expressed and purified as soluble molecules making them ideal candidates for in vitro, biochemical and structural investigations [8, 9, 42, 43]. In the case of the ENTH domain of epsins, the molecular mechanism that leads to protein-induced membrane deformation was widely accepted to depend on the membrane insertion of an amphipathic α-helix (helix 0) that folds upon interaction with PI(4,5)P_2_ [8, 9]. Though many reports are in good agreement with this model, a different explanation for the induction of membrane curvature was recently reported that relies on macromolecular crowding [44, 45]. Our own recent results showed that binding of the ENTH domain helix 0 to membranes results in a decrease in lateral membrane tension [46, 47], which in turn would lead to a decreased membrane bending modulus and results in a membrane more susceptible to deformation [48]. Together with the results presented here it seems reasonable to assume that not only amphipathic helix insertion is necessary for curvature induction but also a tight spatial arrangement of the protein on the membrane’s surface, seen here as oligomerization, contributes to membrane deformation. This is in line with the notion that the ENTH domain together with the related ANTH domain co-assemble into oligomers on membranes [49, 50].

The data presented here, therefore suggest that helix insertion and molecular crowding are not be mutually exclusive but might work together. In such a model, the amphipathic α0 of ENTH domains senses local curvatures in undulating membranes. Helix insertion would lead to increased bilayer asymmetry and a reduced membrane bending modulus. Lipid-dependent protein clustering increases the protein density on the membrane, which in turn could lead to more pronounced macromolecular crowding induced membrane deformation.

## Electronic supplementary material

Below is the link to the electronic supplementary material.

Supplementary file1 (DOCX 1162 kb)
