# Supplementary material for: Cooperativity of membrane-protein and protein–protein interactions control membrane remodeling by epsin 1 and affects clathrin-mediated endocytosis
Source: Cell Mol Life Sci. 2020 Sep 30;78(5):2355–70. doi: 10.1007/s00018-020-03647-z (PMC7966211; doi:10.1007/s00018-020-03647-z)
Supplement: Supplementary file 1 — Supplementary file1 (DOCX 1162 kb) [file 18_2020_3647_MOESM1_ESM.docx]

**Supplementary Figures and Material for**

**Cooperativity of membrane-protein and protein-protein interactions control membrane remodeling by epsin 1 and regulate clathrin-mediated endocytosis.**

*Benjamin Kroppen^1^, Nelli Teske^2^, King F. Yambire^3^, Niels Denkert^1^, Indrani Murkhejee^1^, Daryna Tarasenko^1^, Garima Jaipuria^4^, Markus Zweckstetter^4,5^, Ira Milosevic^3^, Claudia Steinem^2,6,7,*^ & Michael Meinecke^1,6,*^*

^1^ Department of Cellular Biochemistry, University Medical Center Göttingen, Humboldtallee 23, 37073 Göttingen, Germany

^2^ Institute for Organic and Biomolecular Chemistry, University of Göttingen, Tammannstr. 2, 37077 Göttingen, Germany

^3^ European Neuroscience Institute Göttingen – A Joint Initiative of the University Medical Center Göttingen and the Max-Planck-Society, Grisebachstr. 5, 37077 Göttingen, Germany

^4^German Center for Neurodegenerative Diseases (DZNE), Von-Siebold-Str. 3a, 37075 Göttingen, Germany

^5^ Max Planck Institute for Biophysical Chemistry, Am Fassberg 11, 37077 Göttingen, Germany

^6^ Göttinger Zentrum für Molekulare Biowissenschaften – GZMB, 37077 Göttingen, Germany

^7^ Max Planck Institute for Dynamics and Self-Organization, Am Faßberg 17, 37077 Göttingen, Germany

*Correspondence to: mmeinec@gwdg.de & csteine@gwdg.de


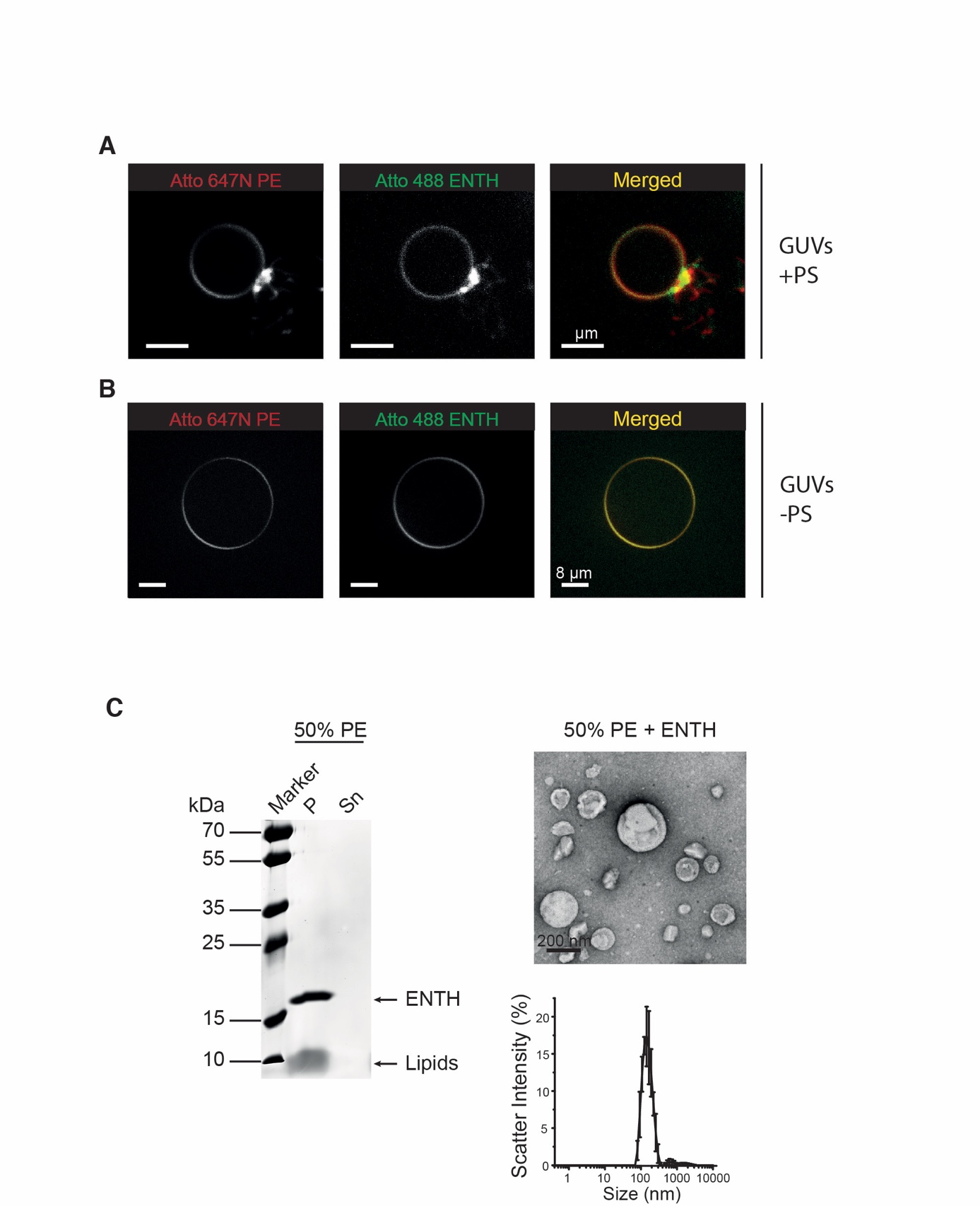


**Supplementary Figure 1:**

**(A)** Membrane binding and deformation by ENTH domains resolved in real time by GUV assays. The ENTH domain is recruited to GUVs and induces membrane deformation on GUVs. After incubation the protein signal (Atto488, green) completely co-localizes at the GUV membranes (Atto847N, red) and membrane tubules are generated on the GUV surface. GUVs were composed of PC, PE, PS, PI(4,5)P_2_ (L-α) and Atto647N DOPE (44.5:30:20:5:0.5 mol%). The ENTH domain was labeled with Atto488-maleimide by cysteine modification with an DoL of 0.98. The scale bars correspond to 8 µm.

**(B)** In contrast, ENTH domain induced membrane deformation was not observed if GUVs did not contain PS, although the protein was still recruited to the GUV surface. GUVs were composed of PC, PE, PI(4,5)P_2_ (L-α) and Atto647N DOPE (64.5:30:5:0.5 mol%). The ENTH domain was labeled with Atto488-maleimide by cysteine modification with an DoL of 0.98. The scale bars correspond to 8 µm.

**(C)** To find out if ENTH domain induced membrane deformation is PS specific or due to changes of physical properties within the membrane, the concentration of unsaturated L-α PE was increased to 50% because it has a similar inverse conical shape. The ENTH domain co-sediments with LUVs consisting of PC PE PI(4,5)P_2_ in spin assays. But in contrast to PS containing membranes the ENTH domain did not form oligomers. No ENTH domain induced membrane deformation was observed on LUVs of this composition by electron microscopy and the DLS scattering profile remained as a sharp peak indicating for no deformation. LUVs used for this spin assay were composed of PC, PE, PI(4,5)P_2_ (45:50:5 mol%).


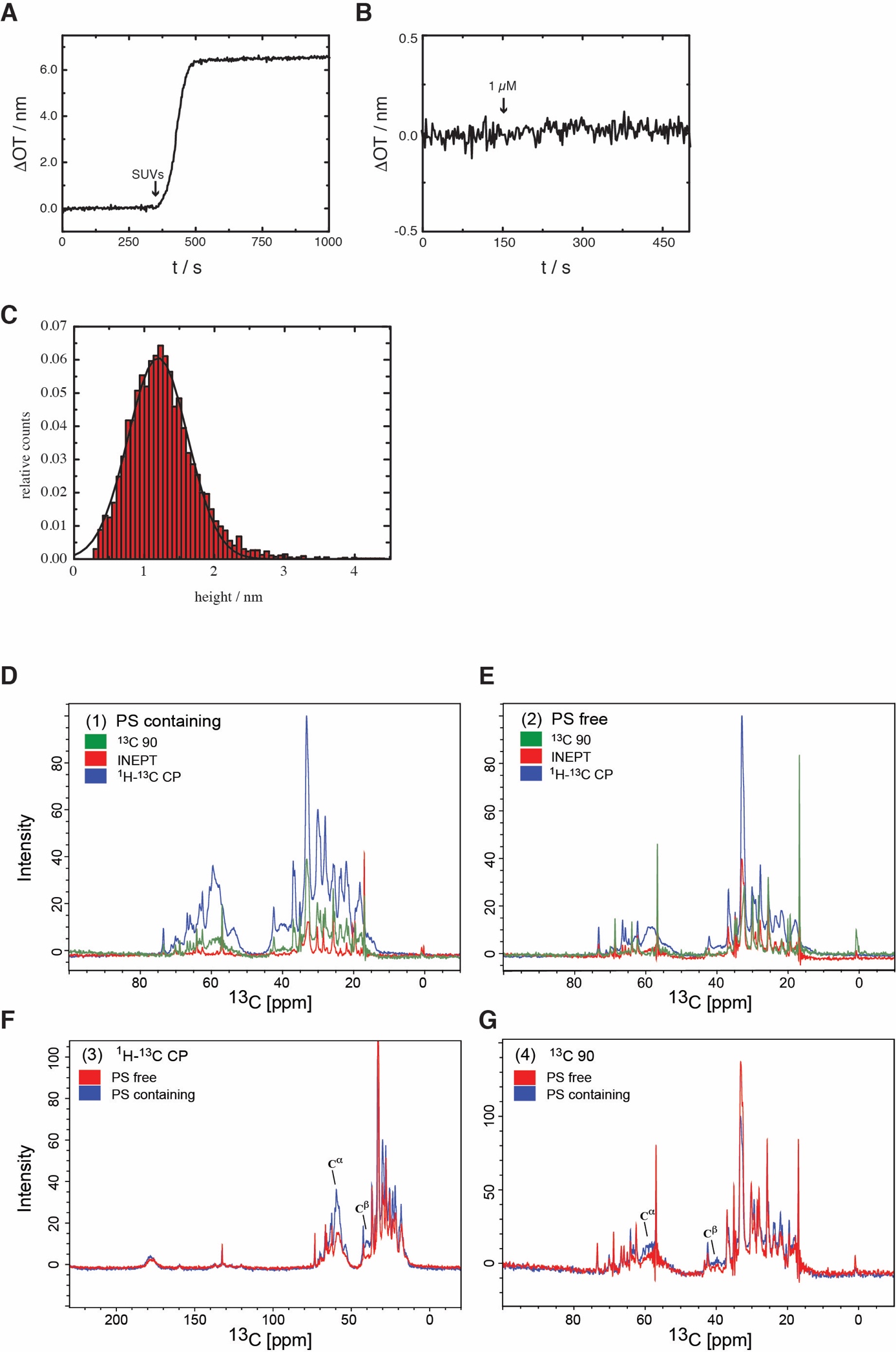


**Supplementary Figure 2:**

(**A**) Time-resolved change in optical thickness upon surface spreading of SUVs.

**(B)** Time-resolved change in optical thickness upon addition of 1 µM ENTH^WT^ to a POPC/POPS (80:20) bilayer, showing no binding of the protein in the absence of PI(4,5)P_2_.

**(C)** Height analysis of AFM image shown in Figure 2M. Fitting a normal distribution resulted in a protein height of 1.2 ± 0.5 nm.

(**D**) Comparison of spectra recorded for ENTH bound to PS containing membrane. ^1^H-^13^C CP (blue) spectrum shows peaks from rigid residues of the protein, INEPT spectrum shows peaks from flexible parts of protein/lipid and ^13^C 90 experiment shows spectrum from all parts of the sample.

(**E**) Comparison of spectra recorded for ENTH bound to PS-free membrane.

(**F**) Comparison of Cα, Cβ regions of protein from ^1^H-^13^C CP experiment for ENTH bound to PS-free and PS containing membrane. Increase in signal intensity in Cα and Cβ regions of ENTH bound to PS containing membrane is an indicator of increased rigidity.

(**G**) Comparison of ^13^C90 spectrum of the protein in PS-free and PS-containing membrane.


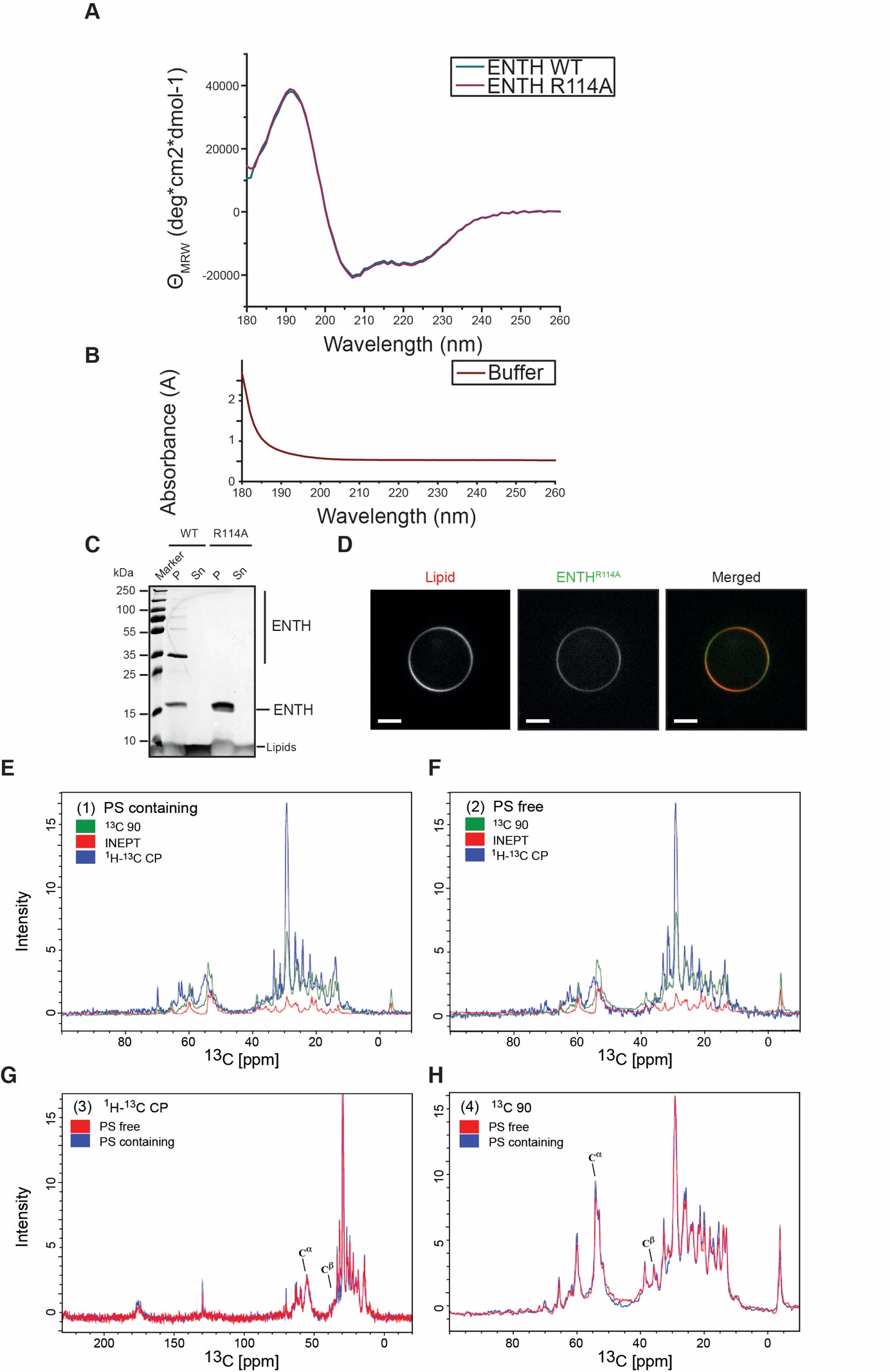


**Supplementary Figure 3:**

**(A)** The CD spectroscopy analysis indicated that the mutation R114A did not induce helix breaks or misfolding in the structure of ENTH^R114A^. ENTH^WT^ and ENTH^R114A^ were transferred to 10 mM potassium phosphate buffer (pH 7.4, 0.1 mg/ml *c*_protein_). The CD spectroscopy analysis of the ENTH domain WT displayed a typical profile of a protein consisting of α-helices, indicating correct folding. In comparison, the CD profile of ENTH^R114A^ displayed a nearly perfect overlay over the profile of the ENTH^WT^. We also measured the absorbance of the potassium phosphate buffer to show that it did not interfere the CD spectroscopy in the range of 190 nm to 260 nm. CD spectroscopic profiles of ENTH^R114A^ (purple) and the ENTH^WT^ (cyan).

**(B)** Absorbance of the potassium phosphate buffer.

**(C)** SDS-PAGE of a spin assay of ENTH^WT^ and ENTH^R114A^ with LUVs containing PC, PE, PS and PIP_2_ (44.5:30:20:5:0.5 mol%).

**(D)** ENTH^R114A^ binds to membranes but fails to induce membrane deformation also in GUV experiments. After incubation the protein signal (Atto488, green) completely co-localizes at the GUV membrane (Atto847N, red) but no deformation or defined protein induced GUV collapse was observed. GUVs were composed of PC, PE, PS, PI(4,5)P_2_ and Atto647N DOPE (44.5:30:20:5:0.5 mol%). ENTH^R114A^ was labeled with Atto488 maleimide by cysteine modification with an DOL of 1.1. The scale bars correspond to 8 µm.

**(E)** Comparison of ^1^H-^13^C CP (blue), INEPT and 13C90 NMR spectrum recorded for ENTH^R114A^ in PS conditions.

**(F)** Comparison of spectra recorded for ENTH^R114A^ in PS-free conditions.

**(G)** Comparison of Cα, Cβ regions of protein from ^1^H-^13^C CP experiment for ENTH^R114A^ bound to a PS-free or PS containing membrane. Similar signal intensity in Cα and Cβ regions from both conditions is an indicator of similar rigidity of samples.

**(H)** Comparison of ^13^C90 spectrum of the protein for ENTH^R114A^ in PS-containing and PS-free membrane.


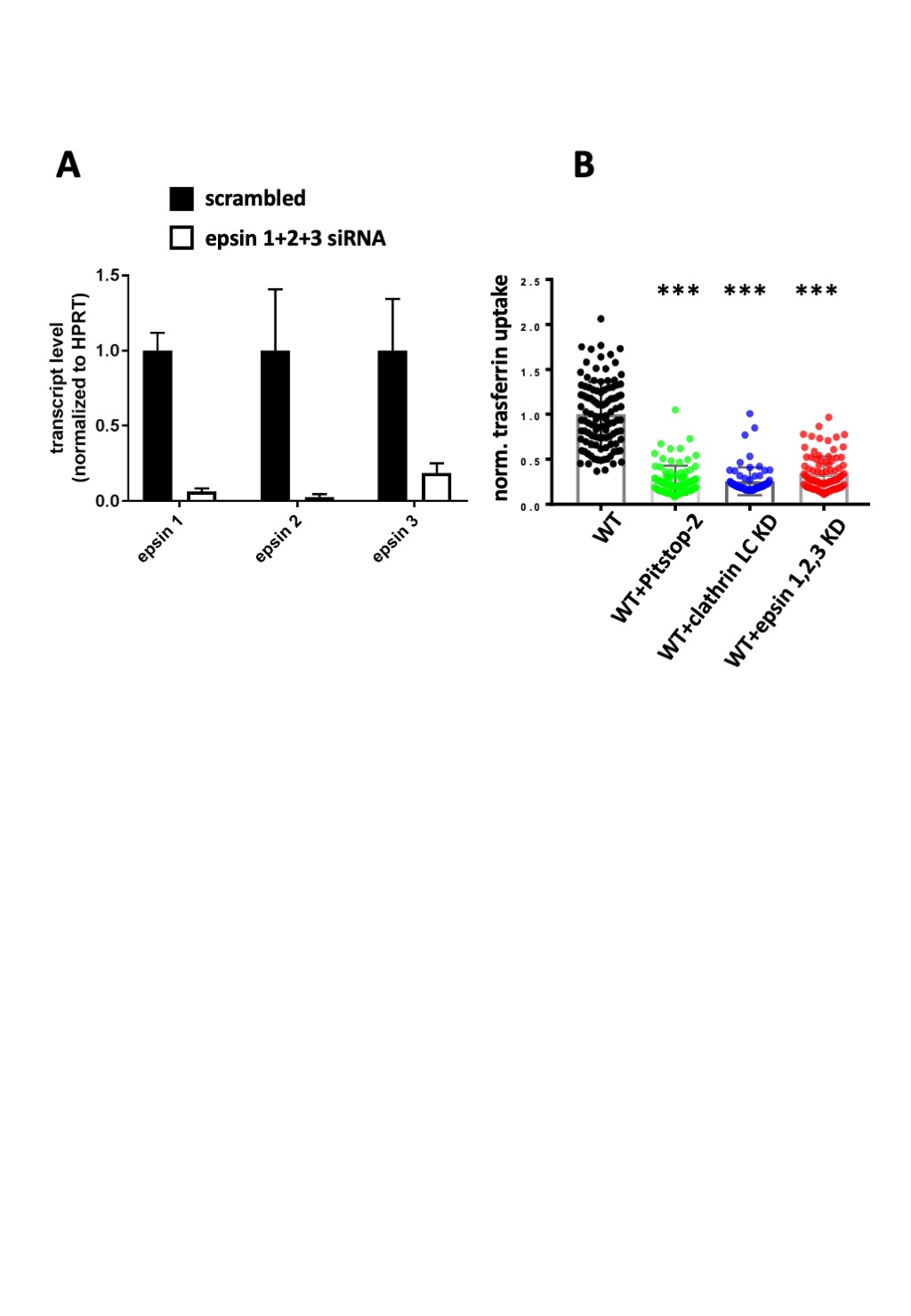


**Supplementary Figure 4:**

**(A)** The transcript levels of three epsin genes (1, 2 and 3) measured by quantitative PCR to inspect the efficiency of epsin 1,2,3 knock-down (KD) in HeLa cells, as in Boucrot et al. (2012). Experiments were performed as detailed in Methods. Three independent experiments, each with technical duplicates, were performed in the case of epsin 1 and epsin 2. Two experiments with technical repetitions were performed for epsin 3. Mean ± SD, three independent experiments were performed. The levels of detected epsins transcripts were significantly lower in HeLa cells with epsin 1,2,3 KD.

**(B)** Analysis of internalized fluorescent transferrin-Alexa Fluor548 in HeLa cells treated with PItstop-2, HeLa cells where clathrin light chain was knocked-down and in HeLa cells where epsin 1, 2 and 3 were knocked-down. Experiments were based on Boucrot et al. (2012), and performed as detailed in Methods. Data are presented as mean ± SD. 3-4 experiments were performed for all conditions. ∗∗∗p<0.001, one-way ANOVA.
